# Phase I study of ADI‐PEG20 plus low‐dose cytarabine for the treatment of acute myeloid leukemia

**DOI:** 10.1002/cam4.3871

**Published:** 2021-03-30

**Authors:** Hui‐Jen Tsai, Hui‐Hua Hsiao, Ya‐Ting Hsu, Yi‐Chang Liu, Hsiao‐Wen Kao, Ta‐Chih Liu, Shih‐Feng Cho, Xiaoxing Feng, Amanda Johnston, John S. Bomalaski, Ming‐Chung Kuo, Tsai‐Yun Chen

**Affiliations:** ^1^ National Institute of Cancer Research National Health Research Institutes Tainan Taiwan; ^2^ Department of Oncology College of Medicine National Cheng Kung University Hospital National Cheng Kung University Tainan Taiwan; ^3^ Division of Hematology/Oncology Department of Internal Medicine Kaohsiung Medical University Hospital Kaohsiung Medical University Kaohsiung Taiwan; ^4^ Division of Hematology Department of Internal Medicine College of Medicine National Cheng Kung University Hospital National Cheng Kung University Tainan Taiwan; ^5^ Division of Hematology‐Oncology Department of Internal Medicine Chang Gung Memorial Hospital at Linkou Linkou Taiwan; ^6^ Division of Hematology‐Oncology and Cancer Center Chang Bing Show Chwan Hospital Changhua Taiwan; ^7^ Polaris Pharmaceuticals, Inc Polaris Group San Diego CA USA

**Keywords:** acute myeloid leukemia, arginine deprivation, low‐dose cytarabine, pegylated arginine deiminase (ADI‐PEG20), phase I

## Abstract

Most acute myeloid leukemia (AML) cells are argininosuccinate synthetase‐deficient. Pegylated arginine deiminase (ADI‐PEG20) monotherapy depletes circulating arginine, thereby selectively inducing tumor cell death. ADI‐PEG20 was shown to induce complete responses in ~10% of relapsed/refractory or poor‐risk AML patients. We conducted a phase I, dose‐escalation study combining ADI‐PEG20 and low‐dose cytarabine (LDC) in AML patients. Patients received 20 mg LDC subcutaneously twice daily for 10 days every 28 days and ADI‐PEG20 at 18 or 36 mg/m^2^ (dose levels 1 and 2) intramuscularly weekly. An expansion cohort for the maximal tolerated dose of ADI‐PEG20 was planned to further estimate the toxicity and preliminary response of this regimen. The primary endpoints were safety and tolerability. The secondary endpoints were time on treatment, overall survival (OS), overall response rate (ORR), and biomarkers (pharmacodynamics and immunogenicity detection). Twenty‐three patients were included in the study, and seventeen patients were in the expansion cohort (dose level 2). No patients developed dose‐limiting toxicities. The most common grade III/IV toxicities were thrombocytopenia (61%), anemia (52%), and neutropenia (30%). One had an allergic reaction to ADI‐PEG20. The ORR in 18 evaluable patients was 44.4%, with a median OS of 8.0 (4.5‐not reached) months. In seven treatment‐naïve patients, the ORR was 71.4% and the complete remission rate was 57.1%. The ADI‐PEG20 and LDC combination was well‐tolerated and resulted in an encouraging ORR. Further combination studies are warranted. (This trial was registered in ClinicalTrials.gov as a Ph1 Study of ADI‐PEG20 Plus Low‐Dose Cytarabine in Older Patients With AML, NCT02875093).

## Graphical abstract

Combination of ADI‐PEG20 at 36 mg/m2 with LDC is tolerable with acceptable toxicities in relapsed/refractory or poor‐risk acute myeloid leukemia and induced an overall response rate of 44.4% and a median overall survival of 8.0 months, The overall response rate and complete remission rate are 71.4% and CR of 57.1%, respectively, in treatment‐naive patients. This regimen suggests the synergistic effect of the combination of ADI‐PEG20 and chemotherapy and is warranted for further study.

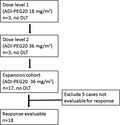

**Abbreviations**
ADI‐PEG20: pegylated arginine deiminase
AML: acute myeloid leukemia
LDC: low‐dose cytarabine

## 1 INTRODUCTION

Acute myeloid leukemia (AML) is a disease characterized by a heterogeneous clonal expansion of immature myeloid cells.^1^ More than 50% of AML cases are diagnosed in patients >60 years.^2^ Intensive chemotherapy induces a high complete remission (CR) rate in AML; however, more than half of these cases typically relapse within 2 years.^3^, ^4^ Extensive genetic aberrations have been identified in AML and have provided important clues for the treatment of AML.^5^ Many novel agents targeting FLT3, IDH1/2, and c‐KIT or multi‐targeted kinase inhibitors alone or in combination with chemotherapy have been shown to increase the response rate and prolong the survival of patients with AML having specific genetic aberrations.^6^, ^7^, ^8^, ^9^, ^10^

Arginine, a semi‐essential amino acid in humans, is not only required for protein synthesis in all tissues but also plays an important role in tumor metabolism.^11^, ^12^ *De novo* arginine is biosynthesized via conversion of citrulline to arginine by argininosuccinate synthase (ASS1) and argininosuccinate lyase. ASS1 is the rate‐limiting enzyme for endogenous arginine production in the urea cycle.^11^ Numerous cancers have been shown to be ASS1‐deficient; hence, cancer cells are dependent on exogenous arginine.^12^ Therefore, arginine deprivation may lead to the death of cancer cells but not of normal cells. Arginine deiminase (ADI) is a mycoplasma enzyme that can deplete arginine and selectively induce the death of tumor cells.^13^, ^14^ ADI‐PEG20 is pegylated arginine deiminase, which can induce the depletion of arginine via rapid conversion of arginine into citrulline, which inhibits the proliferation of various ASS1‐deficient cancer cells in vitro and in vivo.^15^, ^16^, ^17^ High proportions of patients with AML have been shown to be ASS1‐deficient.^18^ Indeed, AML has been described as addicted to arginine.^19^, ^20^ The effects and toxicities of ADI‐PEG20 monotherapy in relapsed/refractory (R/R) or poor‐risk AML have been shown in a phase II study; complete remission was observed in two cases after treatment with ADI‐PEG20, with the response lasting for 7.5 and 8.8 months.^21^

ADI‐PEG20 monotherapy has resulted in modest response rates, not only in AML but also in hepatocellular carcinoma (HCC), metastatic melanoma, and malignant pleural mesothelioma (MPM).^22^, ^23^, ^24^, ^25^, ^26^ However, the combination of ADI‐PEG20 and chemotherapy has augmented the effect of either chemotherapy or ADI‐PEG20 based on in vitro and in vivo studies. Further, these findings have translated into enhanced overall response rate (ORR) and apparent overall survival (OS) in patients with MPM or non‐squamous non‐small cell lung cancer treated with pemetrexed and cisplatin, patients with pancreatic cancer treated with gemcitabine and nanoparticle albumin‐bound paclitaxel, and patients with HCC on a FOLFOX treatment regimen.^27^, ^28^, ^29^ The preclinical biochemical pharmacologic finding that supports improvement in ORR and OS is ADI‐PEG20‐induced downregulation of ribonucleotide reductase M2 (RRM2) found in pancreatic cancer cells, thus enhancing the intracellular concentrations of gemcitabine.^30^ RRM2 catalyzes the conversion of ribonucleotides to deoxynucleotides, a rate‐limiting step in DNA synthesis and repair, and has been associated with clinical outcomes in patients with cancer receiving nucleoside analog‐based chemotherapy.^31^ As cytarabine is another deoxycytidine analog‐like gemcitabine, suppression of RRM2 will also be expected to increase the intracellular concentration of cytarabine alongside its intended effect. In addition, the combination of BCT‐100, a pegylated human recombinant arginase that leads to a rapid depletion of intracellular and extracellular arginine concentrations, with cytarabine has been shown to exert a synergistic effect on the cytotoxicity of AML cells.^20^ The results suggest the potential benefit of combining chemotherapy with arginine‐depleting agents for the treatment of AML.

Thus, based on the prior encouraging results of ADI‐PEG20 combination chemotherapy trials, the previous results of ADI‐PEG20 monotherapy in AML, and the observed enhancement of intracellular nucleoside analog concentrations with the use of ADI‐PEG20, the combination of ADI‐PEG20 with the standard of care low‐dose cytarabine (LDC) was evaluated for its tolerability and efficacy in R/R or poor‐risk AML.

## 2 PATIENTS AND METHODS

### 2.1 Eligibility criteria

This study was sponsored by the Polaris Group and registered with ClinicalTrials.gov as NCT02875093 on Aug 23, 2016. The enrolled patients were diagnosed with AML and >17 years old and had an ECOG performance ≤2. The patients were R/R or poor‐risk AML and unfit for intensive chemotherapy or could not tolerate or access azacitidine or other hypomethylating agents. Patients unfit for conventional intensive chemotherapy were defined as those with advanced age (>75 years), impaired function of organs (heart, lung, liver, and kidney), or any other comorbidity that physicians have judged to be unfit for intensive chemotherapy. The creatinine clearance was ≥30 mL/min, the total bilirubin level was ≤2 × upper limit of normal (ULN), and the AST/SGOT and ALT/SGPT levels were ≤3 × ULN. Patients who had uncontrolled concomitant illnesses, were diagnosed with acute promyelocytic leukemia, had previously received arginine‐depleting agents, or were hypersensitive to cytarabine were not eligible. The study was approved by the institutional review board of each participating institution and was performed in accordance with the guidelines and regulations of each participating hospital.

### 2.2 Treatment and evaluation

This phase I trial was conducted to evaluate ADI‐PEG20 in combination with LDC in a conventional 3 + 3 study design. The primary endpoint was to assess the safety and tolerability of this combinational regimen. The secondary endpoints were time on treatment, OS, ORR, and biomarkers (pharmacodynamics and immunogenicity detection). ADI‐PEG20 (18 or 36 mg/m^2^) was administered weekly via the intramuscular route and cytarabine 20 mg was administered subcutaneously twice daily for 10 days every 28 days (28 days as a cycle). Each new dose level cohort was to be entered 28 days after the last subject was entered in the prior cohort. If two of three patients developed dose‐limiting toxicity (DLT) in dose level 1 (18 mg/m^2^), the dose level of ADI‐PEG20 was reduced to 9 mg/m^2^. An expansion cohort was enrolled at the maximal tolerated dose of ADI‐PEG20 to further assess toxicity in this tumor type and to obtain a preliminary estimate of efficacy. The severity of all adverse events was assessed according to the NCI CTCAE Scale, version 4.0. DLT was evaluated in the first cycle of treatment. Hematologic DLT was defined as pancytopenia with hypocellular bone marrow (BM) and no marrow blasts lasting for ≥6 weeks after the start of a cycle. Grade III/IV non‐hematologic toxicities related to the treatment drugs were defined as DLT except nausea/vomiting/diarrhea, rash, and fatigue with recovery observed within 72 hours, 2 weeks, and 5 days under medical treatment, respectively. The complete blood count (CBC) and differential count were evaluated every week. BM aspiration or biopsy was performed at baseline and then after cycles 2, 3, and at the clinically indicated time as judged by the investigator. The BM aspiration or biopsy could be performed after cycle 1 treatment if there was a significant response noted by CBC data. The tumor response was assessed according to the revised recommendations of the International Working Groups.^32^, ^33^ Disease control rate was defined as the percentage of patients who have achieved complete response, partial response, and stable disease with the use of the specified treatment regimen. Treatment was continued until the following were reported: unacceptable toxicity, death, disease progression, noncompliance, refusal of the patient to continue treatment, decision by the investigator to terminate treatment, or up to 24 cycles of treatment. Patients may receive up to four additional cycles of ADI‐PEG20 when CR, CR with incomplete recovery (CRi), or complete cytologic response (CCR) was reached after treatment.

Measurement of pharmacodynamic and immunogenicity parameters including circulating levels of arginine, citrulline, and antibodies to ADI‐PEG20 were performed on days 1, 8, 15, 22, and 27 of cycle 1, as well as day 1 of cycles 2–6. Circulating arginine and citrulline levels and plasma anti‐ADI‐PEG20 antibody titers were measured using mass spectrometry assays and an ELISA‐based immunogenicity assay, respectively.^21^ Arginine depletion was defined as ≤10 μM of arginine level. The tests for pharmacodynamic and immunogenicity parameters were performed by Polaris Pharmaceuticals, Inc. (San Diego, CA, USA).

### 2.3 Statistical consideration

The primary endpoint was the safety and tolerability of this regimen. The secondary endpoints were time on treatment, OS, ORR, and pharmacodynamics and immunogenicity. The safety, pharmacodynamics, and immunogenicity of ADI‐PEG20 were analyzed according to the intention‐to‐treat (ITT) principle. The ORR and OS were analyzed for the per‐protocol evaluable population. The evaluable patients were those who received at least two consecutive doses of the study drug during the first 2 weeks, had a completed baseline tumor evaluation, and at least one post‐baseline tumor evaluation. Descriptive statistics were used. Data collected as continuous variables were summarized as mean ±standard deviation or median (minimum, maximum). Data collected as categorical variables were summarized by frequency (%). The known ORR for subjects that met the inclusion criteria for this trial was approximately 15–20%. An increase to 35% ORR was considered “encouraging.” Survival distributions were estimated using the Kaplan‐Meier method.

## 3 RESULTS

### 3.1 Patient characteristics and treatment delivery

**FIGURE 1:**
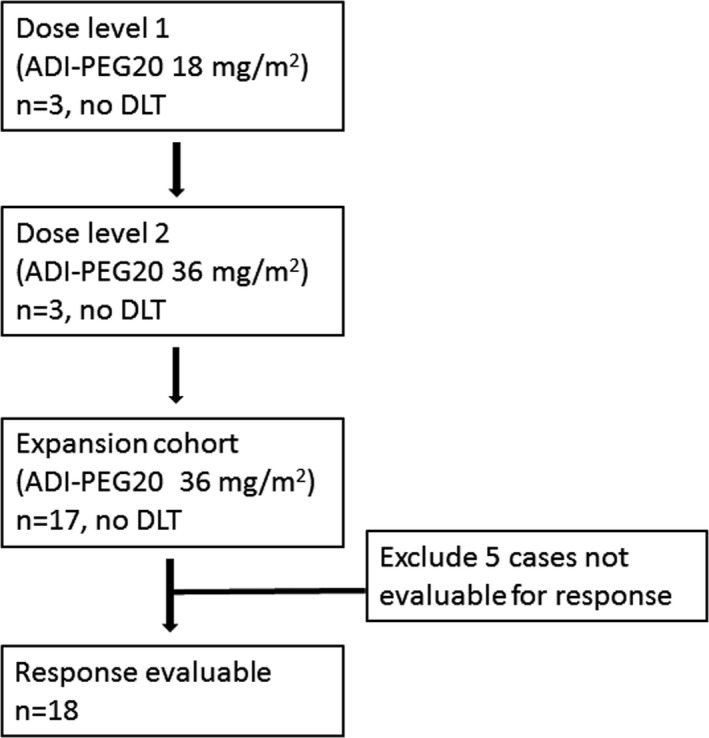
Flow diagram of the enrollment of patients in this study

Twenty‐three patients were enrolled in the study from January 2017 to July 2018, with data cutoff in September 2019. The patient demographics and baseline characteristics are listed in Table 1. The flow diagram of enrollment of the patients in this study is presented in Figure 1.

**TABLE 1:** The demographics and baseline characteristics of the patients

	Cohort 1	Cohort 2 + MTD	Total
	(N = 3)	(N = 20)	(N = 23)
Median Age (range)			66 (33 ~ 80)
Sex, Men			11 (47.8%)
Cytogenetic study			
Normal	0	4 (20%)	4 (17.4%)
Abnormal	3 (100%)	16 (80%)	19 (82.6%)
Risk			
Favorable	1 (33.3%)	0	1 (4.3%)
Intermediate	0	12 (60%)	12 (52.2%)
Adverse	2 (66.7%)	4 (20%)	6 (26.1%)
ASS Expression			
Negative	3 (100%)	19 (95%)	22 (95.7%)
Positive	0	1 (5%)	1 (4.3%)
ECOG Performance Status			
0	0	5 (25%)	5 (21.7%)
1	3 (100%)	12 (60%)	15 (65.2%)
2	0	3 (15%)	3 (13%)
No. of lines of Previous Systemic Treatment			
0	0	9 (45%)	9 (39.1%)
1	0	2 (10%)	2 (8.7%)
2	3 (100%)	1 (5%)	4 (17.4%)
≥3	0	8 (40%)	8 (34.8%)

The median age of the 23 patients was 66 (range: 37–80) years. Sixteen patients (69.6%) were more than 60 years old. There were 11 men and 12 women. Nine patients were treatment naïve, and the other 14 patients were R/R AML. The median age of the nine treatment‐naïve patients was 70 (range: 63–80) years. Eighteen patients were evaluated for tumor response. The remaining five patients were not evaluable for tumor response because they received only one dose of ADI‐PEG20 or did not have a post‐baseline tumor evaluation (BM evaluation). Among these five patients, four patients terminated treatment due to death related to AML. These four patients had only received ≤1 cycle of ADI‐PEG20 and died a few days later after the last dose of ADI‐PEG20. For the ITT analysis, the median duration of treatment was 4.18 (0.03–9.87) months. For the 18 evaluable patients, the median time on treatment was 4.41 (0.69–9.87) months. Eight patients received more than six cycles of treatment. Three patients reported stable disease (SD); these patients discontinued treatment due to withdrawal of consent but did not have progressive disease (PD). These three SD patients received 2, 4, and 11 cycles of treatment, respectively. Two patients discontinued the treatment after the completion of additional four cycles of treatment when developing CR or CRi. One patient discontinued treatment due to the development of anaphylactic shock. The other patients discontinued treatment due to PD.

### 3.2 Safety and tolerability

No patient developed DLT. The maximal tolerated dose of ADI‐PEG20 was 36 mg/m^2^. The treatment‐related toxicities in the ITT population are listed in Table 2. Overall, the most common toxicities were anemia (61%), thrombocytopenia (61%), infection (48%), nausea (48%), and vomiting (39%). Grade III/IV toxicity occurred most frequently in the hematologic system and infection categories, including thrombocytopenia (61%), anemia (52%), neutropenia (30%), febrile neutropenia (22%), and infection (17%). Grade III anaphylactic shock developed in one patient and was attributed to ADI‐PEG20. This patient recovered after treatment for the anaphylaxis, without any sequela.

**TABLE 2:** Treatment‐related adverse events of ADI‐PEG 20 and/or low‐dose cytarabine

Grade	I	II	III	IV	All
anemia	0	2	9	3	14 (61%)
neutropenia	0	0	0	7	7 (30%)
thrombocytopenia	0	0	1	13	14 (61%)
Febrile neutropenia	0	0	0	5	5 (22%)
Infection*	0	7	2	2	11 (48%)
pyrexia	4	2	1	0	7 (30%)
nausea	7	4	0	0	11 (48%)
vomiting	7	2	0	0	9 (39%)
diarrhea	1	2	1	0	4 (17%)
hiccup	1	0	0	0	1 (4%)
abdominal pain/distension	2	3	0	0	5 (22%)
stomatitis/mouth ulceration	0	2	0	0	2 (9%)
rash/erythema	4	3	0	0	7 (30%)
ecchymosis/petechiae	3	1	0	0	4 (17%)
pruritus	1	0	0	0	1 (4%)
eczema	0	2	0	0	2 (9%)
fatigue	5	0	0	0	5 (22%)
asthenia	0	1	0	0	1 (4%)
injection site discomfort/pain	2	0	0	0	2 (9%)
injection site swelling	1	0	0	0	1 (4%)
dyspnea	0	3	0	0	3 (13%)
hemothorax	0	0	1	0	1 (4%)
wheezing	0	1	0	0	1 (4%)
hemorrhage	2	1	0	0	3 (13%)
impaired liver function	4	0	0	0	4 (17%)
hypokalemia	0	0	0	1	1 (4%)
decreased appetite	4	2	0	0	6 (26%)
pain in extremities or trunk	0	3	0	0	3 (13%)
arthralgia	1	0	0	0	1 (4%)
soft tissue necrosis	0	1	0	0	1 (4%)
dizziness	2	0	0	0	2 (9%)
headache	1	0	0	0	1 (4%)
anaphylactic shock	0	0	1	0	1 (4%)
hypersensitivity	0	1	0	0	1 (4%)

* infection, including bacterial, viral, and fungal infection.

### 3.3 Efficacy

**FIGURE 2:**
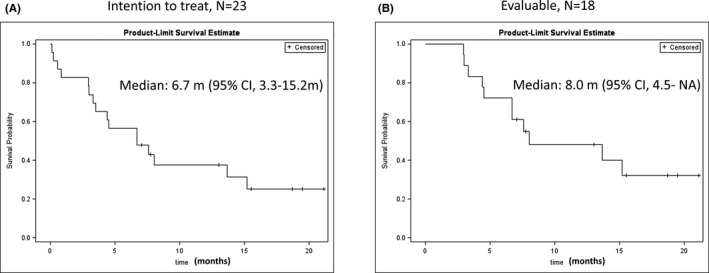
Overall survival of the patients. (A) Intention‐to‐treat population (N = 23). (B) Evaluable population (N = 18)

Among the 23 patients, 18 patients were evaluable for treatment response. Among the 18 evaluable patients, 11 patients were R/R AML, whereas 7 patients were treatment naïve. In the seven treatment‐naïve patients, the ORR was 71.4% and the CR rate was 57.1%. Among the 18 evaluable patients, 7 (38.9%) had CR or CRi and received a 36 mg/m^2^ dose of ADI‐PEG20. Two of these seven patients had CCR. One patient (5.6%) had a PR, and six patients (33.3%) had the best response of SD. The ORR of the 18 evaluable patients was 44.4% (95% confidence interval (CI), 21.5–69.2%). The disease control rate (DCR) was 77.8% (95% CI, 52.4–93.6%). Among the eight responders (CR +PR), six progressed and two patients were still in CR without relapse at the time of data analysis. The median duration of response in the eight responders was 7.9 months (4.4 months–not reached). The median duration to achieve CR or CRi for the seven patients was 12 (range: 8–24) weeks. Among the two patients achieving CR without further relapse, one was a 66‐year‐old woman who had failed six prior lines of treatment before enrollment. She had a CCR after six cycles of ADI‐PEG20 and LDC and stopped the treatment after another four cycles of this regimen. She had a response duration of 18.7 months with persistence of CR. The other patient was an 80‐year‐old man who was treatment‐naïve before enrollment. He achieved CRi after three cycles of this regimen and became CR after additional one cycle of treatment. He stopped treatment at cycle 7 due to the development of anaphylactic shock. The shock recovered after treatment without any sequela; he had a response duration of 7.8 months without relapse at the last final follow‐up date. The median OS for the ITT population was 6.7 (95% CI, 3.3–15.2) months. The median OS for all 18 evaluable patients was 8.0 months (95% CI, 4.5 months–not reached). The OS of the ITT and evaluable patients is shown in Figure 2. For the six patients who achieved SD after treatment, the median treatment duration was 5.3 (range: 1.6–9.9) months. The demographics, treatment, response, and treatment duration of each patient are listed in Table 3.

**TABLE 3:** The demographics, treatment, and treatment outcome of each patient

Case No.	Sex	Age	status at accrual	dose level of ADI‐PEG20*	best response	cause of termination	treatment duration**	OS**	status
1	F	64	R/R	1	SD	Patient withdrawal	1.64	4.51	dead
2	M	42	R/R	1	SD	Investigator decision	9.87	15.20	dead
3	F	55	R/R	2	SD	PD	6.28	7.57	dead
4	F	60	R/R	2	CRi	Relapse	4.38	8.03	dead
5	F	66	R/R	MTD	CCR	CR	9.01	18.72	dead
6	F	63	treatment naïve	MTD	SD	PD	6.22	6.68	dead
7	F	37	R/R	MTD	SD	Investigator decision	3.09	13.68	dead
8	F	67	R/R	MTD	PD	PD	1.91	3.32	dead
9	F	69	treatment naïve	2	CR	relapse	6.32	21.12	alive
10	M	74	treatment naïve	MTD	CR	CR!	6.32	19.47	alive
11	M	64	treatment naïve	MTD	PR	PD	9.05	15.53	alive
12	M	79	R/R	MTD	CR	relapse	4.18	6.68	dead
13	M	80	treatment naïve	MTD	CR	relapse	4.41	7.07	alive
14	F	59	R/R	MTD	PD	PD	1.64	2.99	dead
15	F	70	treatment naïve	MTD	PD	PD	1.68	2.96	dead
16	F	63	R/R	MTD	PD	PD	0.69	4.41	dead
17	M	71	R/R	MTD	SD	PD	4.41	13.03	alive
18	M	80	treatment naïve	MTD	CCR	AE	6.02	7.76	alive

Response rate (all) ORR: 44.4% (CR:38.9%, PR: 5.6%) median OS: 8.0 months (95% CI, 4.5~ not reached)

Response rate (treatment naïve) ORR:71.4% (CR:57.1%, PR:14.3%)

Response rate (R/R) ORR:27.3% (CR:27.3%, PR:0)

* 1: 18 mg/m^2^, 2: 36 mg/m^2^, MTD: 36 mg/m^2^.

** months.!, The patient had relapse of disease during follow‐up after the completion of treatment.

### 3.4 Pharmacodynamic and immunogenicity parameters

**FIGURE 3:**
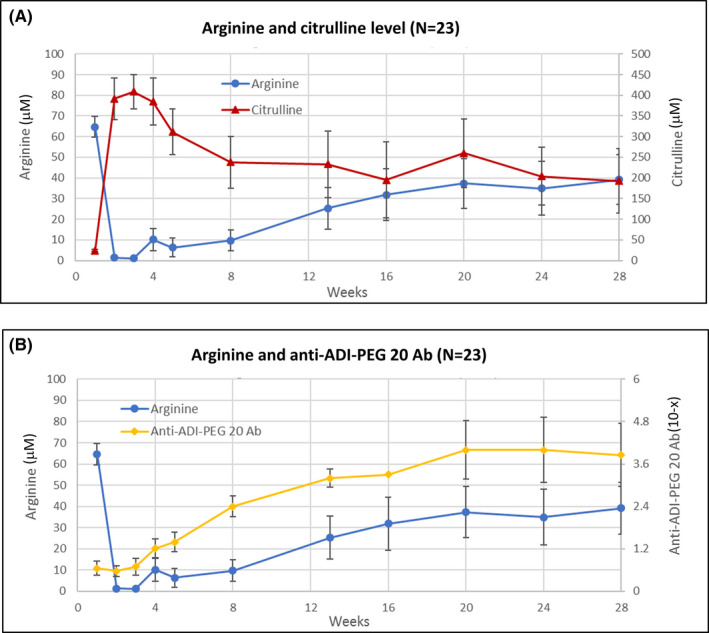
Dynamic changes in arginine, citrulline, and anti‐ADI‐PEG20 antibody levels in the intention‐to‐treat patients. (A) Dynamic change in circulating arginine and citrulline levels. (B) Dynamic change in circulating arginine and anti‐ADI‐PEG20 antibody levels

The mean arginine level of all 23 patients for ITT analysis at baseline was 64.7 ± 5 μM; the arginine level of these patients, except two patients for whom no data are available, was undetectable or less than 10 μM a week after ADI‐PEG20 and LDC treatment. The mean arginine level of the patients remained less than 10 μM for 8 weeks after treatment initiation and increased gradually. The decrease in arginine levels was accompanied by an increase in citrulline levels. An increase was also observed in anti‐ADI‐PEG20 antibody levels in the fourth week after treatment with ADI‐PEG20 and LDC. The dynamic changes in arginine, citrulline, and anti‐ADI‐PEG20 antibody levels in the ITT patients are shown in Figure 3. The mean duration of arginine levels being less than 10 μM or undetectable was 12 (range: 4–28) weeks for the patients who had the best response of CR or PR.

## 4 DISCUSSION

In this study, we showed that the combination of ADI‐PEG20 with LDC was tolerable in 23 R/R or poor‐risk patients with AML. There was one case of anaphylaxis, which has been observed previously with the administration of ADI‐PEG20, as well as with cytarabine.^21^, ^25^, ^34^ Our study included a diverse group of patients by age (young and elderly patients) and treatment (R/R and treatment‐naïve patients). The standard treatment for patients newly diagnosed with AML is induction chemotherapy. For R/R AML, allogeneic hematopoietic stem cell transplantation (HSCT) is considered if there is a donor available and if the patient's condition is suitable for such treatment. There are various chemotherapy regimens that are considered before the initiation of HSCT, such as FLA‐IDA or high‐dose cytarabine combined with mitoxantrone. However, these salvage regimens are intensive. The patients who are not suitable for intensive chemotherapy or allogeneic HSCT are recommended to enroll in clinical trials or consider palliative treatment.^35^ The toxicity of ADI‐PEG20 was tolerable according to previous clinical trials. However, the toxicities of the combination of ADI‐PEG20 with LDC are still not well understood. Therefore, we included a diverse patient population who were not suitable for intensive chemotherapy in our current study. There were nine treatment‐naïve patients in our current study. The median age of the treatment‐naïve patients was 70 years with an age range from 63 to 80 years. They were not suitable for intensive chemotherapy by the principle investigators’ judgment. The diverse patient population may also be one of the factors affecting the toxicities and efficacy of this regimen.

The toxicities of ADI‐PEG20 monotherapy with either 18 mg/m^2^ or 36 mg/m^2^ have been shown to be tolerable with only a few grade 3/4 toxicities noted in previous phase I/II studies for various cancer types.^22^, ^25^, ^36^ Furthermore, in a placebo‐controlled phase III trial, the adverse effects of ADI‐PEG20 monotherapy were similar to those of the placebo.^26^ Similarly, when combined with chemotherapy, the adverse events observed in ADI‐PEG20 triplet studies have been consistent with those of chemotherapy alone.^27^, ^28^, ^29^ ADI‐PEG20 monotherapy has been shown to be tolerable in R/R or poor‐risk patients with AML in this study except for the development of anaphylactic shock in one case.^21^ Except for grade III/IV anemia and thrombocytopenia, other toxicities of the combination of LDC and ADI‐PEG20 in our study were not worse than the toxicities observed in patients with AML who received LDC alone.^37^, ^38^, ^39^, ^40^ It is difficult to compare the toxicities reported in our current study with those in previous studies of LDC alone for patients with AML due to differences in baseline characteristics. Although the percentages of grade III/IV anemia (52%) and thrombocytopenia (61%) were higher in our current study, the percentages of neutropenic fever and infection were not higher than those reported by the previous studies using LDC alone. One possible cause of higher grade III/IV hematologic toxicities in our study is that the patients in our study had intensive medication regimens and only nine (39%) patients were treatment naïve, whereas the patient populations in previous studies were mostly treatment‐naïve elderly patients with AML.^37^, ^38^, ^39^, ^40^ Except in a phase III placebo‐controlled trial,^26^ anaphylactic shock due to ADI‐PEG20 monotherapy has been observed previously.^21^, ^25^ This event is attributed to ADI being a nonhuman protein and thus warrants caution for toxicity although the prevalence has been low, especially when compared to pegylated asparaginase, another anti‐cancer enzyme therapy.^41^ Our current study showed the tolerability of the combination of ADI‐PEG20 with LDC for R/R or poor‐risk patients with AML.

In a phase II trial using ADI‐PEG20 alone for R/R or poor‐risk patients with AML, two responders (9.5%) with prolonged OS (17.1 and 16.9 months) were noted among 21 evaluable patients.^21^ Similarly, the response rate has been generally low in other prior monotherapy ADI‐PEG20 trials.^22^, ^23^, ^24^, ^25^, ^26^, ^36^ Many factors may contribute to the low response rate of ADI‐PEG20 monotherapy in various cancers. Development of resistance to ADI‐PEG20 via the upregulation of ASS1 or metabolic reprogramming after prolonged arginine depletion was reported to be a possible reason.^42^, ^43^, ^44^ Development of antibodies against ADI‐PEG20 was also reported as a possible reason for ADI‐PEG20 resistance in clinical settings.^45^ Nevertheless, the response rate increased significantly when combined with chemotherapy compared to that with chemotherapy alone.^20^, ^27^, ^28^, ^29^, ^30^, ^31^ LDC is a commonly used standard treatment for either untreated or treated patients with AML who are not suitable for induction or intensive chemotherapy. The response rate of LDC ranged from 2.3% to 34%, whereas the median OS of LDC for patients with AML ranged from 4.5 to 5.6 months.^38^, ^39^, ^40^, ^46^, ^47^ Indeed, in our current study, the combination of LDC with ADI‐PEG20 was shown to have an ORR of 44.4% and a median OS of 8.0 (4.5–not reached) months among the 18 evaluable patients. Hence, the result seems better than that with ADI‐PEG20 monotherapy for R/R or poor‐risk AML.^21^ The efficacy of this regimen was also better than that of LDC alone for elderly patients with AML in previous studies, although it is difficult to compare our study with previous studies due to different baseline characteristics.^37^, ^38^, ^39^, ^40^, ^46^, ^47^ However, our patient population had intensive medication regimens, with 61% and 35% of patients having received ≥1 and ≥3 lines of treatment before enrollment, respectively. The results suggest the potential benefit of this regimen for R/R or poor‐risk patients with AML. In addition, the ORR of 71.4% and CR of 57.1% in the seven treatment‐naïve patients obtained in our study correlates well with the results observed in the registration studies of glasdegib +LDAC at 26.9% (ORR) and 8.8 months (OS), respectively^37^ and with venetoclax +LDAC at 54% (ORR) and 10.1 months (OS), respectively.^48^ Both these studies included untreated patients. The results from the current study demonstrate the efficacy of the combination in the treatment of AML.

Development of anti‐ADI‐PEG20 antibodies alongside an elevation in arginine levels was considered a possible reason for the treatment failure of ADI‐PEG20 in patients with ASS1‐deficient cancer. In the current study, we found that the median duration of arginine depletion is 8 weeks, which was longer than the median duration of 2 weeks for arginine depletion in patients with AML treated with ADI‐PEG20 alone.^21^ In addition, the time for the development of anti‐ADI‐PEG20 antibodies in the current study was significantly delayed compared to that in the AML trial using ADI‐PEG20 alone.^21^ Although the exact mechanism is not clear, the results suggest that the combination of ADI‐PEG20 with LDC may delay the development of anti‐ADI‐PEG20 antibodies and prolong the duration of arginine depletion for patients with AML, in a manner similar to cytarabine decreasing antibodies against asparaginase in children with ALL.^49^ This prolonged suppression of peripheral blood arginine and anti‐ADI‐PEG20 antibody with ADI‐PEG20 + LDAC is consistent with that observed with other chemotherapies when combined with ADI‐PEG20.^27^, ^28^, ^29^

In conclusion, a weekly dose of 36 mg/m^2^ ADI‐PEG20 in combination with LDC is tolerable with acceptable toxicities. This regimen in R/R or poor‐risk AML induced an ORR of 44.4% and a median OS of 8.0 months among the 18 evaluable patients and an ORR of 71.4% and CR of 57.1% in the 7 treatment naïve patients. This regimen suggests the synergistic effect of a treatment regimen that utilizes a combination of ADI‐PEG20 and chemotherapy with acceptable toxicities.

## CONFLICTS OF INTEREST

This trial was sponsored by the Polaris Group. All authors declared no financial conflicts of interest, except for those who are Polaris Group employees Xiaoxing Feng, Amanda Johnston, and John S. Bomalaski are employees of the Polaris Group.

## Data Availability

The data that support the findings of this study are available on request from the corresponding author. The data are not publicly available due to privacy or ethical restrictions.
